# Quality of life: modified triple-branched stent graft implantation versus frozen elephant trunk technique

**DOI:** 10.1186/s13019-021-01683-9

**Published:** 2021-10-13

**Authors:** Zeng-Rong Luo, Mi-Rong Tang, Jia-Hui Li, Liang-Wan Chen, Liang-Liang Yan

**Affiliations:** 1 Department of Cardiovascular Surgery and Cardiac Disease Center, Union Hospital, Fujian Medical University, Fuzhou, 350001 People’s Republic of China; 2 Key Laboratory of Cardio-Thoracic Surgery (Fujian Medical University), Fujian Province University, Fuzhou, People’s Republic of China

**Keywords:** SF-36, Quality of life, Self-perceived burden scale, Aortic surgery

## Abstract

**Objective:**

To compare the effects of modified triple-branched stent implantation and frozen elephant trunk technique on the quality of life (QoL) of acute Stanford Type A aortic dissection (AAAD) patients at different follow-up times.

**Methods:**

Data from 175 AAAD survivors was collected which were divided into two groups according to different surgical techniques: (group A): modified triple-branched stent graft implantation; (group B): frozen elephant trunk. The SF-36 were used to assess the QoL at discharge (AD), the third postoperative month (POM3), and the twelfth postoperative month (POM12).

**Results:**

(1) The total scores at each time of both groups showed lower than the normal level; Group A scored higher than group B at some time points in terms of some items (role physical, role emotion and mental health; all P = 0.000), and some items at POM3 or POM12 scored higher than at discharge (role physical, social function; both P = 0.000). (2) There were less patients with heavy self-perceived burden in group A than group B at discharge (P = 0.032) and patients with heavy self-perceived burden decreased over time. (3) Young postoperative AAD patients (P = 0.002) in group B (P = 0.005) with heavy self-perceived burden (P = 0.000), acute renal failure (P = 0.008), long LOS (P = 0.026) and blood loss (> 1000 mL/24 h) (P = 0.039) seemed to get a worse QoL.

**Conclusion:**

The impact on QoL of the modified triple-branched stent graft implantation technique seemed to be better than those of frozen elephant trunk surgery in role physical, role emotion and mental health.

## Introduction

Acute type A aortic dissection (AAAD) is a seriously critical and complicated disease which is usually associated with various possible complications. Without proper surgical intervention approximately 75% of patients decease within two weeks after the oncoming of symptoms [1]. Surgery is the most effective treatment method. Postoperative mortality and complications have been considered to be a important indicators of surgical treatment effect. Many previous studies concerns about postoperative outcome, patient survival rates, and complications resulting in disability [1, 2]. Surgical outcome has obviously improved due to constant progress in surgical technique, anesthesia technology and medical therapy [3]. However, with constant efforts to achieve patient-centric health care, increasing attention has been paid to more subjective assessments such as quality of life (QoL), health status (HS), and patient satisfaction (PS) [4]. We performed this research to assess the QoL after different surgical techniques for arch and descending dissection in AAAD patients at different follow-up period.

## Patients and methods

### Patients data

By respectively setting alpha value to 0.05 and statistical power value to 0.9 and conducting a power analysis with Gpower 3.1.9, we found that this study should include at least 60 patients [5]. Therefore the number of patients was set to 100. Between January 2018 and December 2019, we finally conducted a questionnaire survey of 200 AAAD survivors who underwent emergency surgery of the arch and descending dissection, 100 of whom use modified triple-branched stent graft implantation technique [6] in arch and descending dissection, 100 of whom use frozen elephant trunk technique [7]. According to different techniques, we divided the patients into two groups that were respectively named Group A (modified triple-branched stent graft implantation technique) and Group B (frozen elephant trunk technique). All the surgeons was proficient in both surgical techniques and the patients were assigned to surgical techniques according to the surgeon’s preference with the patients’ informed consent. Inclusion criteria: ① diagnosed with AAAD by aortic computed tomography angiography (CTA); ② age over 18 years; ③ consent to inclusion into our institution's prospective aortic registry; ④ surviving the procedure and undergoing a standardized follow-up protocol according to the university's interdisciplinary board for aortic disease for at least one year. Exclusion criteria: ① had a history of mental illness; ② combined with other diseases that significantly affected the quality of life, such as malignant tumors; ③ unable to hear and speak; ④ refused to cooperate with the investigation. The study was approved by the Hospital Ethics Committee Evaluation criteria.

### Measuring quality of life

We used the Medical Outcomes Trust, 36-Item Short Form Health Survey (SF-36), a survey with established overall reliability and validity, to evaluate patient’s QoL [8–10]. Because it is comprehensive and concise, it is more and more widely used at home and abroad [11, 12]. The SF-36 consists of 36 questions, grouped into eight multiple-item domains, including physical functioning, role physical, bodily pain, general health, vitality, social functioning, role emotional and mental health, [13]evaluating different aspects of daily life. We scored according to the results of the SF-36 questionnaire and respectively got the total score of these eight aspects.

Self-Perceived Burden Scale was developed by Cousineau et al. [14], with a total of 10 items, using a Likert 5 score with a total score of 10–50. The content of scale included 10 aspects the patients worried and concerned about the inconvenience and burden to the caregivers. And there were five answers about how often you feel this way: None of the time, A Little of the time, Some of the time, Most of the time, All of the time; the corresponding score was 1 point, 2 points, 3 points, 4 points, 5 points. In addition, the higher the total score on this scale, the heavier the patient feels the burden; the total score is graded by 20 points. A score ≤ 20 means that the patient has no obvious perceived burden, 21 to 30 points means a mild burden, 31 to 40 points means a moderate burden, and > 40 points means a heavy burden. The total score is divided into 2 subgroups: No obvious burden or mild burden were regarded as no impact group, and moderate burden and heavy burden were regarded as impact group.

### Baseline characteristics

Baseline characteristics of the patients were collected through historical records, preoperative patient medical reports, physical examinations and clinical examinations.

### Surgical methods

All procedures were performed through right axillary arterial intubation and bilateral antegrade selective cerebral perfusion under circulation at moderate body temperature. Aortic root operations were then conducted, including reconstruction of the aortic sinus, Bentall procedure, Wheat procedure, and coronary artery bypass grafting based on the direct exploration of the involvement of aortic root. The arch and descending aortic dissection were found in patients. Depending on the different surgical techniques for arch and descending dissection, the patients was divided into two groups: (Group A) was defined as a patient underwent modified triple-branched stent graft implantation technique; (Group B) were defined as patients underwent frozen elephant trunk technique. The process is documented [6, 7].

### Data collection

The first author and investigator of this study used a unified survey. Instead of using inductive and suggestive words, a questionnaire was collected and recorded on the spot to check the integrity of the data. Questionnaires were collected on the morning at discharge, the third postoperative month and the twelfth postoperative month.

### Statistical methods

Data analysis was manipulated using SPSS23.0 software. Normality test on measurement data was conducted firstly. Measurement data in accordance with normal distribution were expressed as mean ± standard deviation, count data were expressed as composition ratio (%); Repetitive general linear model followed by the Bonferroni correction was used to compare the different procedures. We applied Greenhouse–Geisser correction to intersubjective effect. The time-dependent changes in SF-36 scores were analyzed by Friedman's test. To examine where the differences actually occurred, the Wilcoxon signed-rank test was performed on the different groups. Two-category variables were analyzed by single factor analysis using t-test to satisfy multi-classification of homogeneity of variance; multiple linear regression was conducted for multivariate analysis; P < 0.05 was regarded as statistically significant.

## The result

### Baseline characteristics

In this study, a total of 200 cases met the inclusion criteria, 25 cases did not complete follow-up(4 case of paralysis following acute stroke, 1 case of metastatic cancer, 3 case on dialysis, 14 patients refused to finish questionnaire survey, 3 patients were lost to follow-up), and the remaining 175 cases were devided into Group A (95 patients) and Group B (80 patients). The baseline characteristics of the patients are listed in Table 1. There was no statistical difference in baseline characteristics between the two groups.

**Table.1:** Preoperative characteristics

	Total	Group A	Group B	*P*
Patients (n)	175	95 (54.3%)	80 (45.7%)	–
Age (years)	52.8 ± 11.8	53.9 ± 11.4	50.1 ± 12.4	.919
Male (n%)	138 (78.9%)	73	65	.477
Diabetes (n%)	29 (16.6%)	16	13	.916
Hypertension (n%)	143 (81.7%)	76	67	.523
Hyperlipidemia (n%)	33 (18.9%)	18	15	.973
Coronary heart disease (n%)	10 (5.7%)	4	6	.349
Renal dysfunction^a^ (n%)	40 (22.9%)	18	22	.180
COPD (n%)	10 (5.7%)	4	6	.349
Moderate or severe aortic valve regurgitation (n%)	61 (34.9%)	30	31	.321
LVEF (%)	62.1 ± 9.5	62.3 ± 11.6	64.1 ± 9.9	.230
Heart rate	85.8 ± 14.5	80.9 ± 13.9	86.5 ± 14.2	.390
Malperfusion syndromes (n%)	26 (14.9%)	13	13	.634

Group A: modified triple-branched stent graft implantation technique, group B: frozen elephant trunk technique

LVEF, left ventricular ejection fraction; COPD, chronic obstructive pulmonary disease

^a^Defined as preoperative creatinine greater than 1.5 mg/dl

### Intraoperative and postoperative results

The total average operation time was 310.6 ± 64.8 min. Compared with group B, the operation time in group A was significantly shorter (290.5 ± 87.8 and 346.6 ± 50.5 min, respectively, P = 0.030). Due to aortic regurgitation, 12.0% underwent aortic valve repair, 15.4% underwent aortic valve replacement, with no significant difference between the two groups. In addition, there were no significant differences in bleeding(> 1000 mL / 24 h), double thoracotomy to stop bleeding, secondary tracheal intubation, neurologic dysfuction, acute kidney injury, hepatic insufficiency, multiple organ dysfunction syndrome, and length of hospital stay (LOS) between the two groups. Table 2 lists the detailed intraoperative and postoperative results.

**Table.2:** Intraoperative and post-operative data of patients in group A and group B

	Total	Group A	Group B	*P*
Intraoperative				
Patients (n)	175	95(54.3%)	80(45.7%)	–
Operation time (min)	310.6 ± 64.8	290.5 ± 87.8	346.6 ± 50.5	.030
Cardiopulmonary bypass (min)	171.4 ± 68.2	141.8 ± 35.6	196.5 ± 45.86	.001
Crossclamp time (min)	80.3 ± 54.8	48.9 ± 18.8	98.4 ± 25.3	.071
Selective cerebral perfusion and low body arrest time (min)	20.1 ± 7.6	14.1 ± 4.6	39.6 ± 15.5	.034
Aortic valve repair (n%)	21(12.0%)	12	9	.779
Aortic valve replacement (n%)	27(15.4%)	20	27	.059
Post-operative				
Bleeding(> 1000 mL/24 h) (n%)	24(13.7%)	9	15	.076
Double thoracotomy to stop bleeding (n%)	5(2.9%)	1	4	.180
Secondary tracheal intubation (n%)	9(5.1%)	4	5	.734
Neurologic dysfuction^a^ (n%)	8 (4.6%)	4	4	–
Acute kidney injury^b^ (n%)	53 (30.3%)	12	11	.827
Hepatic insufficiency^c^ (n%)	54 (30.9%)	28	26	.666
Multiple organ dysfunction syndrome (n%)	5 (2.9%)	3	2	–
Hospital time (days)	19.3 ± 7.8	18.6 ± 13.8	19.5 ± 12.7	.277

Group A: modified triple-branched stent graft implantation technique, group B: frozen elephant trunk technique

^a^Defined as coma, delayed awakening, disorientation, convulsions, hemiplegia, severe limb muscle dysfunction, etc.

^b^Defined as 50% rise in baseline creatinine or new need for dialysis

^c^Defined as bilirubin greater than 5 mg/dL persisting for more than 5 days postoperatively

### Quality of life

**Fig. 1:**
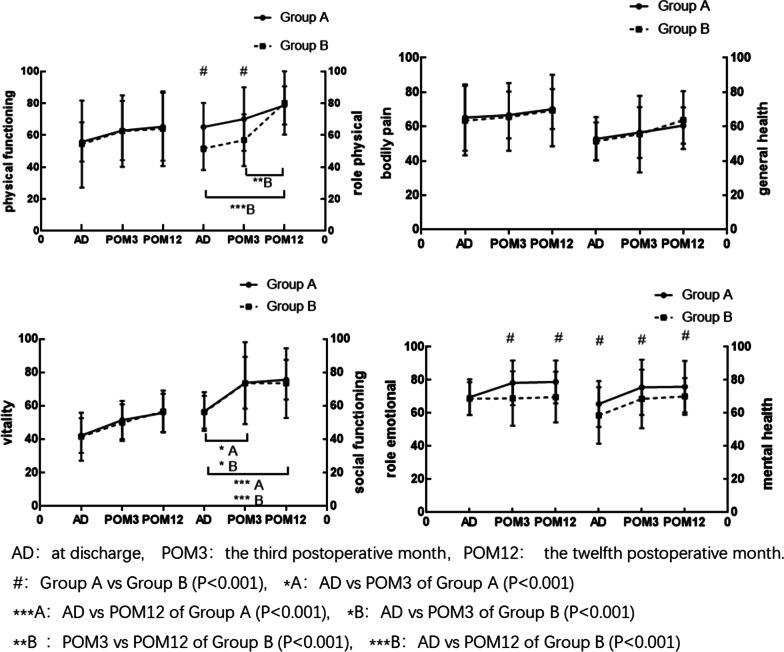
The SF-36 scores of patients using modified triple-branched stent graft implantation or frozen elephant trunk technique at different follow-up times. Group A: modified triple-branched stent graft implantation technique, Group B: frozen elephant trunk technique

SF-36 questionnaire. Figure 1 shows us the SF-36 scores of patients using modified triple-branched stent graft implantation or frozen elephant trunk surgical technique at different postoperative time points. (1)Among the all eight specific projects of the SF-36, the scores at each time point (at discharge, POM3, and POM12) were all below normal levels. (2)With respect to physical functioning, social function, vitality, bodily pain, and general health, we found no statistically significant difference between the two groups at each time point (at discharge, POM3, and POM12). (3)With respect to role physical, the score was higher in group A than in group B at discharge (P = 0.000) and POM3 (P = 0.000), but no significant difference was found between group A and group B at POM12 (P = 0.279). The score at POM12 in group B was higher than that at discharge (P = 0.000) and at POM3 (P = 0.000), but no significant difference was found between score at discharge and at POM3 (P = 0.566). (4) With respect to social function, the score at POM3 (P = 0.000, 0.000 respectively) and at POM12 (P = 0.000, 0.000 respectively) of two groups were both higher than that at discharge, but no significant difference was found between score at POM3 and at POM12 (P = 0.360, 0.804 respectively). (5) With respect to role emotion, we found no significant difference between the two groups at discharge, and the score of group A was higher than that of group B at POM3 (P = 0.000) and POM12 (P = 0.000). (6) With respect to mental health, group A obtained higher score than group B at any time point (P = 0.000, 0.000, 0.000 respectively).

### Self-perceived burden scale

**Fig. 2:**
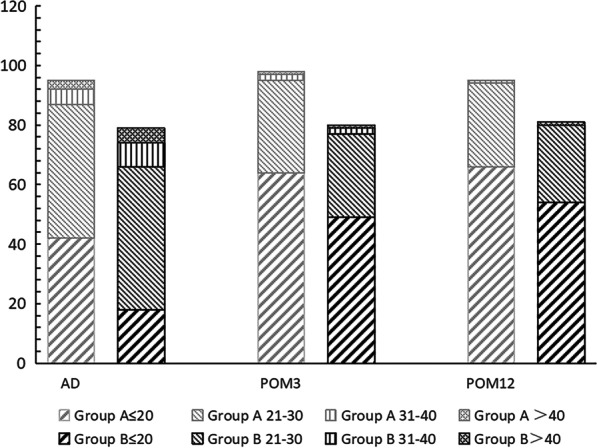
The different distribution of self-perceived burden score between modified triple-branched stent graft implantation and frozen elephant trunk technique at different follow-up times. Group A: modified triple-branched stent graft implantation technique, Group B: frozen elephant trunk technique

The patients’ Self-Perceived Burden distribution of two groups at different time points is shown in Fig. 2. A total score ≤ 20 means that the patient has no obvious perceived burden, 21 to 30 points means a mild burden, 31 to 40 points means a moderate burden, and > 40 points means a heavy burden. The total score is divided into 2 subgroups: No obvious burden or mild burden were regarded as no impact group, and moderate burden and heavy burden were regarded as impact group. From Fig. 2, we found that group A showed significant difference in self-perceived burden at different follow-up times: as the postoperative time increased, patients influenced by the self-perceived burden gradually decreased, as did Group B. We also found a statistically significant difference at discharge between the two groups: at discharge, the influence of the self-perceived burden of group A was less than that of group B (P = 0.032). However, we found no significant difference between the two groups at POM3 and POM12.

### Multiple linear regression analysis of patients' quality of life

A multiple linear regression analysis of the interfering factors related to QoL was performed one year after discharge. As shown in Table 3, taking the average total score of the SF-36 scale as the dependent variable, preoperative characteristics, intraoperative and post-operative data and self-perceived burden were used as independent variables for multiple linear regression analysis.The result showed that there was a linear regression relationship between the QoL of patients and group, self-perceived burden, age, acute renal failure, LOS, blood loss (> 1000 mL/24 h). Young patients with heavy self-perceived burden, acute renal failure, long LOS and blood loss (> 1000 mL/24 h) in group B seemingly obtained lower QoL.

**Table.3:** Multiple linear regression analysis of patients' quality of life

Variable	Unstandardized B	Coefficients std. error	Standardized coefficients β	t	P
Constant	1.016	0.239		3.267	0.000
Group A	0.997	0.056	0.659	3.875	0.005
Self-perceived burden	− 0.275	0.034	− 0.739	− 8.117	0.000
Age	0.197	0.093	0.535	5.099	0.002
Acute renal failure	− 0.389	0.289	− 0.496	− 4.095	0.008
Length of stay	− 0.487	0.172	− 0.337	− 3.479	0.026
Blood loss (> 1000 mL/24 h)	− 0.985	0.071	− 0.106	− 1.872	0.039

Group A: modified triple-branched stent graft implantation technique

## Discussion

Surgery for AAAD is one of the most complicated and challenging clinical problems in adult cardiac surgery, especially when the arch and descending aortic segment is involved, although outcomes of surgery for AAAD have improved [15]. Some surgeons propose a radical surgical repair principle that try to remove and graft the arch and descending aortic dissection in order to achieve a better mid-term and long-term effects. In-hospital mortality rate is reported in the range of about 0–10%, depending on the acute type A aortic dissection, emergency, average age and the incidence of Marfan’s syndrome [16–20]. Accordingly, more than 90% 1-year survival rate of approximately 80% and 10-year survival rate can be attained [16, 17, 20]. With the development of surgical methods, in addition to disability and mortality, surgeons recently pay increasing attention to conduct analysis of patient-centered treatment results, driven largely by health-care policymakers, payers, and surgical associations [21]. QoL may be of increasing significance in the treatment of patients with acute AAAD. The quality of life can comprehensively assess the overall health status of patients, and it has been paid more and more attention as a feedback indicator. Quality of life is defined as an individual’s concept of life related to the social and cultural values of the society he lives in and base on his goals, expectations, standards and concerns by The World Health Organization (WHO) [22–24]. For patients with AAAD, there is no questionnaire for the disease. In this study, SF-36 was implemented because it was regarded as the most suitable means of measuring QoL for cardiovascular patients [4], although the patients spend longer time answering questions.

In our study, we found the total SF-36 scores of the both groups at each time point (at discharge, POM3, and POM12) were all below normal levels, especially at discharge, which is consistent with the findings of foreign scholars Ghazy et al. [25, 26] This may be because AAAD is an emergency and progresses rapidly, accompanied by severe chest and back pain, and even coma, waist and abdominal pain, and severe surgical trauma. AAAD demonstrates poor short-term prognosis, even providing timely surgical treatment [27, 28]. At discharge, due to the impact of impaired self-care ability, unstable condition, high hospital costs, incidence of postoperative complications, the patient is in a strong stress state, patients easily feel overburdened and have poor QoL after surgery. The constant clicking of mechanical valves are also regarded as a facilitating factor leading to annoyance, disturbance, sleeping disorders, inattention and social embarrassment in some cases of aortic mechanical valve replacement, which will also increase the self-perceived burden feelings and reduce the QoL [29]. While, some items of SF-36 scores seemed to pick up (at POM3, POM12) and the number of patients influenced by the self-perceived burden gradually decreased over time, which might be due to improved cardiac function, improved physical condition and adaptation to surgery.

The results of this study also suggested that: at discharge, the influence of the self-perceived burden of group A was less than that of group B; regarding some specific QoL items, group A may have a higher QoL advantage: role physical, the score was higher in group A than in group B at discharge and POM3; mental health, group A obtained higher score than group B at any time point; role-emotion, the score of group A was higher than that of group B at POM3 and POM12. These results may be due to lighter surgical blow in group A because of the shorter cardiopulmonary bypass time, aortic cross-clamp time, selective cerebral perfusion and lower body arrest time in modified triple-branched stent graft implantation technique [6].

The multiple linear regression analysis results showed that younger age, longer LOS and heavier self-perceived burden in group B corresponds to lower QoL score. The analysis may be due to different educational levels. Different ages have different cognition of diseases and the ability to accept medical information. The results of the study by Endlich et al. [28] show that the QoL of young patients is worse than that of older patients, may be because younger patients often become unemployed or have to find a new job and require job retraining, uncommon social environment and life stress, which lead to heavier self-perceived burdens and the decay of QoL.

## Limitations

This study has a major limitation of low statistical power result from the small sample. QoL is a “partial subjective” index that is difficult to assess and highly individual [30]. And it is very difficult to obtain preoperative QoL evaluation in this critical situation. At the same time, there are many factors, such as education level, family income and self-care ability of patients may be involved in affecting the quality of life. Therefore, we might overcome such limitations by expanding the sample size under multi-center cooperation.

## Conclusion

The AAAD postoperative patients were vulnerable to feeling self-perceived burden and obtained lower SF-36 scores than normal, however, this situation seems to have gradually improved over time. Regarding some specific QoL items, modified triple-branched stent graft implantation technique may have an advantage. Young postoperative AAD patients using frozen elephant trunk technique with heavy self-perceived burden, acute renal failure, long LOS and blood loss(> 1000 mL/24 h)seemingly obtained lower QoL in some items.

## Data Availability

Data sharing not applicable to this article as no data sets were generated or analyzed during the current study.

## Abbreviations

QoL: Quality of life
AAAD: Acute type A aortic dissection
LOS: Length of stay
COPD: Chronic obstructive pulmonary disease
